# Progress in Fevers

**Published:** 1903-06-20

**Authors:** 


### PROGRESS IN FEVERS.

**Malaria.**—Speaking to the medical students of Glasgow University on the subject of malaria, Sir William MacGregor emphasised, in the first place, the great importance of the subject to us, with our vast colonial possessions, "subject to the tyranny of the destructive giant malaria, who bestrides the globe and exacts his yearly tribute of scores and scores of thousands of human lives from white and black indiscriminately." In India alone the mortality from fever among the natives in 1900 was 4,919,591, or half a million more than the total population of Scotland. The brilliant researches of Ross, based on the reasoning of Laveran and Manson, had solved the problem of malaria. The changes constituting the "cycle of Ross," one of the marvels of the age, were briefly described. The parasites, small amœboid bodies, entering the red blood corpuscles, convert the hæmoglobin into melanin. In from one to three days they mature into (*a*) asexual forms which, multiplying by segmentation and sporulation, fill the blood with fresh spores, lead to fresh accession of fever and renew the attack on fresh red corpuscles; or (*b*) into male and female gametocytes which, circulating in the blood, are drawn into the stomach of the mosquito by puncture and there undergo development into hundreds of young filamentous blasts which pass into the insect's salivary glands and are again conveyed by puncture into the human circulation. It was pointed out that in Lagos the mortality from malaria was very high among infants, and that 50 per cent. of apparently healthy children, the majority of whom were under two years of age, harboured malarial parasites in their blood. The flat and swampy nature of Lagos intensified the difficulty of combating the disease, but efforts were being made on the lines of Koch, who advocated prophylactic dosing with quinine (10 grains with or without 5 grains of antifebrin may be given once or twice a day), on the lines of Celli, who relies chiefly on the use of mosquito netting and wire netting; and on the lines of Ross, who aims more especially at destroying the mosquito and her breeding places. Sir William expressed his personal belief in the malarial origin of black water fever, for the treatment of which the various cassia remedies, *cassia beareana*, and *cassia occidentalis*, deserved careful investigation. The most successful treatment he knew of was that of the saline enemata or injections advocated by Dr. Gouzien, late of Dahomey. The careful education of native and European alike in the sanitation of malaria was being attempted. The importance of education is also urged by Major Ross,[2] who, speaking recently at Aberdeen, laid similar stress on the immense number of victims claimed by this scourge among children, and in India, and on the immense importance of the subject in relation to commercial and colonial policy. Captain James,[3] in conjunction with Stephens and Christophers, has published some valuable observations upon malaria and mosquitoes in India. Anopheles Rossii, the most abundant species of mosquito, does not appear to become infected naturally, anopheles fluvialis and anopheles culicifacies are the commonest conveyers of malaria. The Indian anopheles may be classed in three subdivisions for purposes of identification, showing :—(1) one-banded palpi; (2) palpi with four white bands; (3) palpi with three white bands. Each species has its own favourite kind of breeding place. As in Africa so in India, a high percentage of children under ten, 60 to 75 per cent., harbour the parasite. Many children apparently healthy really show temperature and malaise towards night. The use of a mosquito net by night, and the wearing of high boots or putties towards evening, to prevent mosquito bites, would make residence safe even in highly malarious districts. Quinine and mosquito nets have not stamped out the disease in Ismailia,[4] however, where Ross found large numbers of anopheletes breeding in ponds and pools which had not been filled in nor treated with petroleum. Ross here advised the destruction of culex as well as anopheles since the varieties found, culex pipiens and steyomigia fasciata were capable of propagating elephantiasis and yellow fever. Two foreign observers, Galli-Valerio and Rochaz,[5] of Lausanne, have been making investigations relative to the hibernation of the larvæ of anopheles and culex. Contrary to the opinion of most authorities, they find many eggs of both varieties survive even severe frost and ice, and that the larvæ will hatch out after considerable chilling and drying when supplied with warmth and moisture. In Texas malaria is very prevalent, one person in 12 contracting the disease, according to Holdert,[6] who finds a solution of tobacco in kerosene oil (one pound to 10 gallons) a more effective destroyer of mosquito larvæ than plain kerosene or that containing camphor or naphthalin. J. T. Moore[7] urges caution in operating in a possibly malarial patient unless the operation is urgent. The presence or absence of malaria should be first determined, and the condition previously treated if found. Ross' new method of microscopical diagnosis[8] is rapid and simple. About 20 cmm. of blood are lightly spread with a needle on a glas slide. The film is dried, and then covered by an aqueous solution of eosin for 15 minutes. After gentle washing, the film is covered with a weak solution of methylene blue for a few seconds. It is then again washed and examined dry or mounted in Canada balsam.

[1] Brit. Med. Jour., Dec. 20. [2] Ibid., Dec. 6, p. 1800. [3] Lancet, Feb. 7, p. 389. [4] Ibid., Feb. 28, p. 621. [5] Jour. of Trop. Med., Jan. 1. [6] Amer. Jour. Med. Sci., March. [7] Med. Rec., Feb. 21. [8] Brit. Med. Jour., Dec. 20, p. 1891.

*(To be continued.)*

